# Determining Simple and Effective Cost Functions for an Efficient Volumetric-Modulated Arcs-Based Stereotactic Radiosurgery for Single Brain Metastases Using Monaco® Planning System

**DOI:** 10.7759/cureus.70560

**Published:** 2024-09-30

**Authors:** Kazuhiro Ohtakara, Kojiro Suzuki

**Affiliations:** 1 Department of Radiation Oncology, Kainan Hospital Aichi Prefectural Welfare Federation of Agricultural Cooperatives, Yatomi, JPN; 2 Department of Radiology, Aichi Medical University, Nagakute, JPN

**Keywords:** brain metastasis, cost function, dose conformity, dose distribution, dose gradient, dose inhomogeneity, multileaf collimator, stereotactic radiosurgery, tumor location, volumetric-modulated arc therapy

## Abstract

Introduction

Volumetric-modulated arcs (VMA) can produce dose distributions suitable for stereotactic radiosurgery (SRS) with a multi-leaf collimator (MLC) for brain metastases (BMs). The treatment planning and verification for VMA are more complicated than for dynamic conformal arcs. The longer the preparation time from image acquisition to the start of irradiation, the higher the risk of tumor growth and/or displacement. This planning study aimed to exploit the simple and effective cost function (CF) for establishing semi-automatic efficient VMA optimization for SRS of single BMs.

Materials and methods

The study population included 30 clinical BMs with a gross tumor volume (GTV) of 0.72-44.30 cc (median 9.81 cc) and a depth of 20-79 mm (median 41 mm). The treatment platform included a 5-mm leaf-width MLC Agility^®^ (Elekta AB, Stockholm, Sweden) and a planning system Monaco^®^ (Elekta AB). Among various physical and biological CFs available, three combinations consisting of just two or three physical CFs were compared. The Target Penalty CF was uniformly used for ensuring the GTV dose. Three different CF combinations were applied for reducing the surrounding tissue doses: (1) the Conformality alone with the 4-cm margin around target (MAT) that optimizes the limited voxels around the GTV (_wo_QO); (2) the Conformality with the 4-cm MAT and the Quadratic Overdose (_w_QO_4 cm); and (3) the Conformality with the 8-cm MAT that optimizes the overall voxels around the GTV and the Quadratic Overdose (_w_QO_8 cm). The prescribed dose was uniformly assigned to each GTV *D*_V-0.01 cc_, the minimum dose of GTV minus 0.01 cc.

Results

Adding the Quadratic Overdose (_w_QO_4 cm and _w_QO_8 cm) significantly improved the overall dose distribution in comparison to the _wo_QO, while no significant difference was observed between the _w_QO_4 cm and _w_QO_8 cm overall. However, for the GTVs of ≥14 cc, the GTV dose conformity and dose gradient outside the GTV boundary, including the dose attenuation margin, were significantly superior in the _w_QO_8 cm than _w_QO_4 cm. In addition, for the GTV depth of ≥41 mm, the GTV dose conformity and the dose concentric lamellarity at 2 mm outside the GTV were significantly superior in the _w_QO_8 cm than _w_QO_4 cm. Meanwhile, for the GTVs of ≥10 cc, the GTV dose was significantly more inhomogeneous in the _w_QO_4 cm than the _w_QO_8 cm. In addition, for the GTVs of <10 cc and the depth of ≤40 mm, the dose concentric lamellarity at 4 mm inside the GTV surface was significantly higher in the _w_QO_4 cm than the _w_QO_8 cm.

Conclusions

Applying at least three physical CFs to a GTV and the head surface contour is recommended as an effective and efficient optimization method using Monaco for VMA-based SRS of single BMs. In addition, optimizing the overall voxels around the GTV is suitable for reducing the surrounding tissue dose, especially for large and deeply located lesions. Templating the combination of the three CFs with the detailed settings allows for semi-automated and rapid treatment planning, facilitating the prompt start of irradiation after image acquisition.

## Introduction

Stereotactic radiosurgery (SRS) is a sine qua non local treatment option with either a palliative or radical aim for brain metastases (BMs) [1]. The number of cases for which complete local remission of BMs is achieved by SRS alone is increasing [2,3]. To attain long-term local control and safety, it is necessary to maintain the biologically effective dose (BED) at the target boundary above a certain level by flexibly increasing the dose fractionation, even for a large symptomatic lesion and/or the lesion localized in an eloquent area [2-4]. BMs can exhibit a variety of pathologies at the brain-tumor interface, that is, the degree of microscopic brain invasion [2,5,6]. The dose distribution should also be tailored to the pathological condition inferred from image findings to ensure sufficient coverage of the microscopic brain invasion with the minimum required dose [2,5,6].

Our guiding principle lately for the dose distribution is to have steep dose gradients inside and outside a gross tumor volume (GTV) boundary [2,7]. Specifically, we have actively tolerated an extremely inhomogeneous GTV dose with the ultra-high dose in the center to prioritize the degree of dose conformity and the steepness of gradient outside the GTV boundary within the range that is physically achievable for each modality [2-4,7]. The concentrically layered steep dose increase inside the GTV boundary is usually undetrimental, rather likely leads to better tumor response, and has therefore been actively adopted [3,7-9]. In practice since 2018, the priority has been to maintain a dose equivalent to the BED ≥80 Gy to the GTV boundary, not to the ≥1-mm margin-added planning target volume (PTV) periphery [2-4,10]. We have also ensured an appropriate dose attenuation margin with the BED ≥50 Gy at ≥2 mm outside the GTV boundary [2,6,10]. This multi-fraction SRS scheme can enhance the treatment efficacy and safety for large, critically located, and/or radioresistant lesions for which conventional practices are insufficiently effective and/or safe [2,4,10,11].

In cases with a large symptomatic lesion concomitant with a massive surrounding edema and remarkable deformation of the surrounding brain parenchyma, it is desirable to initiate SRS as soon as possible after image acquisition [2,3,10,11]. The longer the preparation time after image acquisition, the higher the risk of tumor growth and/or displacement, which inevitably leads to a decline in irradiation accuracy [12]. Furthermore, when applying more than five fractions to ensure safety for large lesions, it is necessary to confirm whether there is significant shape change and/or displacement of the lesion during treatment, by acquiring images available for treatment planning [2,13-16]. Prompt adaptive re-planning is required if significant changes are observed [16]. In frame-based SRS, irradiation is usually initiated within 3-24 hours after image acquisition since the start of its clinical application. However, depending on the modality and irradiation method, the treatment preparation may require more than two to three days, and if over a weekend, it will take more than five days [2,17].

In linac-based SRS using a multileaf collimator (MLC) even with a leaf width of 5 mm, volumetric-modulated arcs (VMA) can produce dose distributions suitable for SRS of BMs [4,7,15,17]. An arc arrangement including at least two non-coplanar arcs (NCAs) with sufficient rotations of 180º-360º is more suitable for VMA-based SRS than more limited rotations appropriate for dynamic conformal arcs (DCA) [18]. However, VMA-based SRS usually requires more days to prepare because the treatment planning and verification are more complicated than those for DCA [17]. Therefore, efficient optimization of the dose distribution is a prerequisite for initiating irradiation within 24 hours of image acquisition. Monaco^®^ (Elekta AB, Stockholm, Sweden), a planning system capable of VMA optimization, allows the use of a variety of physical and biological cost functions (CFs) that make it difficult to choose [19-23]. However, there are very limited reports regarding specific CF selection and the detailed settings suitable for SRS of BMs [20,23]. In the previous planning study, we reported a simple optimization method that defined only a GTV and used only two physical CFs, the Target Penalty and Conformality [7]. The Quadratic Overdose CF can further improve dose conformity to the GTV boundary [20,24]. In addition, the Conformality CF has an option to specify the range of voxels to optimize for efficiently reducing the dose outside a target volume (TV) by selecting either 4 cm or 8 cm of the margin around target (MAT); however, it is unclear which one is optimal [7,24].

This study was conducted to explore simple and effective CF exploitation for semi-automatic efficient VMA optimization using the Monaco system for SRS of single BMs. Specifically, we examined the significance of adding the Quadratic Overdose and the influence of the voxel range for optimization on the dose distribution. This study is part of the efforts to establish a template for VMA-based SRS planning to facilitate the prompt start of irradiation after image acquisition [21].

## Materials and methods

**Figure 1:**
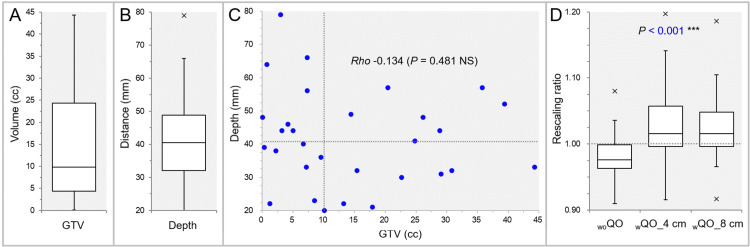
Correlation between gross tumor volume and its depth, and differences in rescaling ratios to align prescription doses after optimization. The images show box-and-whisker plots (BWPs) (A,B,D) and a scatter plot (C); distributions of gross tumor volumes (GTVs) (A) and the depths (B); a correlation between the GTVs and the depths along with the result of Spearman’s rank correlation coefficient (SRCC) (C); and BWPs of the rescaling ratios to equalize the GTV marginal dose and coverage in three different cost function (CF) selection and settings along with the result of Friedman’s test (FT) for comparison. The dotted lines in C and D show the median value of each variable (C) and the 1.000 value meaning no need for rescaling (D), respectively. GTV: gross tumor volume; NS: not significant; _wo_QO: without Quadratic Overdose; _w_QO_4 cm; with Quadratic Overdose with 4 cm margin around target (MAT); _w_QO_8 cm; with Quadratic Overdose with 8 cm MAT.

This was a planning study for the clinical scenarios of single BMs approved by the Clinical Research Review Board of Kainan Hospital Aichi Prefectural Welfare Federation of Agricultural Cooperatives (20220727-1). Thirty lesions were extracted from 27 cases in which multi-fraction SRS was performed previously, and each of the 30 lesions was treated as a single brain metastasis (BM). The GTV was defined as described previously [2,7,24], and each was contoured based on non-contrast-enhanced computed tomography (CT) images (voxel size 0.98 x 0.98 x 1 mm), T2-weighted images (WIs), and contrast-enhanced T1-WIs using a dedicated software MIM Maestro^®^ version 7.1.3 (MIM Software Inc., Cleveland, OH, USA). The depth of the GTV was measured as the distance from the GTV center to the nearest head surface. The descriptive statistics for the GTVs and the depths are shown in Figure 1A,B.

A scatter plot was created to find any correlation between the GTVs and the depths (Figure 1C).

In this study, the prescribed dose was unified as 43 Gy in five fractions, almost equivalent to the BED of 80 Gy, based on the linear-quadratic formula with an alpha/beta ratio of 10, although this dose has been limited to a diameter of ≤3 cm and a GTV of <10 cc in actual clinical practice [2,3]. The dose of 43 Gy in five fractions was assigned to the GTV *D*_V-0.01 cc_, the minimum dose to cover a GTV minus 0.01 cc (*D*_>95%_ for GTV >0.20 cc and *D*_95%_ for GTV ≤0.20 cc), instead of a common fixed percentage coverage of a TV, to reduce the GTV below the prescribed dose to less than 3 mm diameter, as described previously [24,25]. The GTV coverage values with the *D*_V-0.01 cc_ ranged from 95.00% to 99.98% (median value: 99.90; interquartile range [IQR]: 99.77, 99.96) [24].

The treatment platform employed was a 160-leaf and 5-mm leaf-width MLC Agility^®^ (Elekta AB, Stockholm, Sweden) mounted in a linac Infinity^®^ (Elekta AB, Stockholm, Sweden) with a flattening filter-free mode of a 6 MV X-ray beam with the maximum dose rate of 1400 monitor units per minute [26]. The planning system for optimizing VMA-based SRS plans was Monaco^®^ version 5.51.10 (Elekta AB, Stockholm, Sweden) [19-21,23,24]. Each irradiation isocenter was set at the GTV center. Three arcs were uniformly arranged for each GTV, consisting of one coplanar arc with 360º rotation and the collimator angle 0º and two NCAs with each 180º rotation, the collimator angles of 45º and 90º, and the couch rotations of 60º clockwise and counterclockwise, respectively, which are allocated to evenly divide the cranial hemisphere, based on the previous study [18,24]. Double arcs of reciprocating rotation were not allowed. The increment mechanical limiting parameter that controls the number of generated sectors was uniformly set to 20º.

The optimization of VMA-based SRS plans was performed in the Pareto mode with priority given to the steepness of dose falloff outside the GTV [6,7,24]. Three different CF selections and settings compared are shown in Table 1.

**Table 1:** A comparison of the three different cost function selections and settings. *The option of the Multicriterial Optimization is to continue to drive normal tissue sparing while maintaining the target dose. **In the option of the Shrink Structures, including a GTV and adding a margin of 0.2 cm means that the Quadratic Overdose cost function (CF) will work for areas more than 2 mm outside the GTV boundary. CF: cost function; _wo_QO: without Quadratic Overdose; _w_QO_4 cm; with Quadratic Overdose with 4 cm margin around target (MAT); _w_QO_8 cm; with Quadratic Overdose with 8 cm MAT; *D*_V-0.01 cc_: the minimum dose to cover a target volume (TV) minus 0.01 cc (*D*_>95%_ for TV >0.20 cc, *D*_95%_ for TV ≤0.20 cc); RMS: root mean square; GTV: gross tumor volume.

Structure and CF	_wo_QO	_w_QO_4 cm	_w_QO_8 cm
Structures	GTV, Body contour (Patient)		
Target Penalty CF	Prescription (Gy): 43.000 Gy; Minimum Volume (%): 95.00-99.98% (*D*_V-0.01 cc_)		
Conformality CF	Relative Isoconstraint: 0.01 (minimum value); Multicriterial*: +		
Margin around target	4 cm	4 cm	8 cm
Quadratic Overdose CF	-	+ (Maximum Dose: 43.000 Gy; RMS Dose Excess: 0.020 Gy; Multicriterial*: +; Shrink Structure**: GTV, Margin 0.20 cm)	

In this study, the three different CF selections and settings were referred to as the _wo_QO (without Quadratic Overdose), _w_QO_4 cm (with Quadratic Overdose with 4 cm MAT), and _w_QO_8 cm (with Quadratic Overdose with 8 cm MAT), respectively.

**Figure 2:**
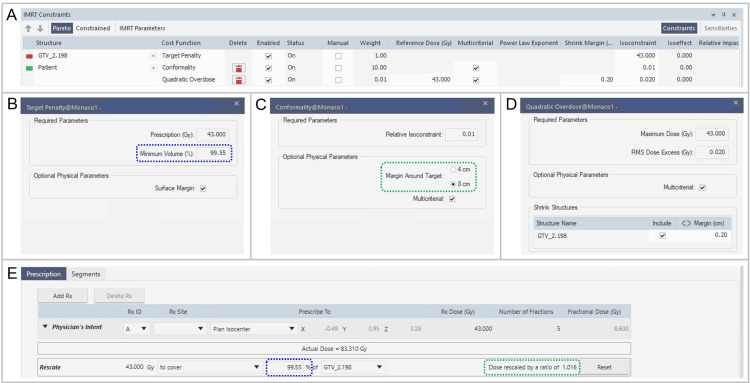
Actual screens for selecting and setting the cost functions applied and for aligning the prescription doses in Monaco system. The images show actual screens for selecting the structures and CFs (A), for setting the Target Penalty (B), Conformality (C), and Quadratic Overdose (D) CFs, and for rescaling the GTV coverage with the prescribed dose to equalize the GTV marginal dose and coverage (E) in Monaco^®^ treatment planning system. (A-E) An example of assigning 43 Gy in 5 fractions to the isodose surface covering 99.55% of the 2.198 cc GTV (GTV_2.198), with optimization using three CFs including the Quadratic Overdose with the 8 cm margin around target (MAT) (_w_QO_8 cm). (B) The coverage value corresponding to the *D*_V-0.01 cc_ for each GTV is entered in the minimum volume field surrounded by the blue dotted line. (C) The area surrounded by the green dotted line determines whether the MAT is 4 cm or 8 cm. (E) After optimization, the same coverage value as the Target Penalty is input into the blank box surrounded by the blue dotted line, and if there is a dose change of 0.1% or more, the rescaled ratio is displayed in the area surrounded by the green dotted line. In the physician’s intent, the dose prescription site is displayed as the irradiation isocenter; however, it is irrelevant to the optimization for volumetric-modulated arc therapy, and our actual intention is sufficient coverage of the GTV boundary by a prescribed dose. CF: cost function; GTV: gross tumor volume; _w_QO_8 cm; with Quadratic Overdose with 8 cm MAT; *D*_V-0.01 cc_: the minimum dose to cover a target volume (TV) minus 0.01 cc (*D*_>95%_ for TV >0.20 cc, *D*_95%_ for TV ≤0.20 cc).

Figure 2 demonstrates the example of actual configuration screens on Monaco system for the _w_QO_8 cm optimization.

The Target Penalty CF was uniformly applied to cover the GTV with the prescription dose, in which the minimum volume (%) was set as the coverage value corresponding to each GTV *D*_V-0.01 cc_ (95.00%-99.98%) (Figure 2B) [24]. The Conformality and Quadratic Overdose CFs were included to ensure the GTV dose conformity and to minimize the dose to the surrounding tissue. The differences between the three methods are as follows. The _wo_QO consists of the Target Penalty and Conformality CFs alone, while the _w_QO_4 cm and the _w_QO_8 cm have the Quadratic Overdose CF added (Figure 2D). The _w_QO_8 cm has the MAT of 8 cm in the Conformality CF (Figure 2C), while the other two methods have that of 4 cm.

The segment shape optimization was included in the sequencing parameters for VMAT with the high-precision leaf positions of the value of 20 for prioritizing the plan quality rather than the speed. Other parameters were uniformly set as the maximum control points of 1024 per arc, the minimum segment width of 0.5 cm, and the medium fluence smoothing. The dose calculation, including tissue heterogeneity correction, was based on the X-ray voxel Monte Carlo algorithm (XVMC) with the dose deposition to the medium setting. The grid spacing and statistical uncertainty per calculation were uniformly set as 0.2 cm and 3.0%, respectively, to prioritize the time savings for optimization. After the optimization, the grid spacing was changed to 0.1 cm and the final dose calculation was performed. After the final dose calculation, each GTV coverage with 43 Gy was rescaled according to each coverage value (≥95%) corresponding to the GTV *D*_V-0.01 cc_ (Figure 2E) [24]. A change in the GTV dose after rescaling of the GTV coverage by the prescribed dose was recorded to the third decimal place as the rescaling ratio, which was displayed as “Dose rescaled by a ratio of X” on Monaco (Figure 2E).

For dosimetric comparisons, isotropic margins of 2 mm, -2 mm, and -4 mm were added to each GTV boundary using MIM Maestro to generate the GTV + 2 mm, GTV - 2 mm, and GTV - 4 mm structures, respectively [6,8]. In general, qualitative evaluation and comparison of plans for SRS were performed using metrics based on the specific fixed coverage of a PTV including a various margin, such as *D*_98%_, *D*_50%_, and *D*_2%_, and relative ratios such as conformity and gradient indices [25,27-30]. Although *D*_98%_, *D*_50%_, and *D*_2%_ may be optimal to express the characteristics of the dose-volume histogram (DVH) curve for a TV, dose prescription to the *D*_98%_ of a GTV or PTV likely leads to a decreasing coverage with the prescribed dose and thereby declining local tumor control with increasing GTV [24]. Therefore, in this study, dosimetric comparisons were performed using metrics more closely relevant to local treatment efficacy and safety [18,24].

An irradiated isodose volume (IIV) was defined and recorded as the volume irradiated with more than a certain relevant dose, including the GTV [18,24]. The IIVs of 100 (prescribed isodose volume, PIV), 75, and 50% of the GTV *D*_V-0.01 cc_ were calculated from the DVH for the volume generated by adding an isotropic 10-30 mm margin to each GTV boundary [18,24]. The absolute volumes obtained by subtracting the GTV from these IIVs were recorded as each spillage volume. The GTV near-maximum dose was recorded as the *D*_0.01 cc_ for GTV ≥0.20 cc or *D*_5%_ (*D*_<0.01 cc_) for GTV <0.20 cc [24], instead of the *D*_2%_ that was deemed inappropriate based on examples such as the *D*_2%_ for GTV of 44.30 cc being the minimum dose of 0.886 cc, equivalent to a 11.9 mm diameter sphere [25].

The GTV dose inhomogeneity was recorded as the GTV *D*_V-0.01 cc_ (%) relative to the GTV near-maximum dose (100%) as defined above [24]. The near-minimum and representative marginal doses of the GTV, GTV + 2 mm, GTV - 2 mm, and GTV - 4 mm structures were evaluated as each *D*_eIIV_ (eIIV: equivalent IIV), which was defined as the minimum dose to cover the IIV equivalent to each TV on the DVH to avoid the substantial over- or under-coverage by the reference dose [6,8]. Each *D*_eIIV_ was recorded as the relative % dose to the GTV *D*_V-0.01 cc_ (100%). The coverage value of each reference TV by the *D*_eIIV_ reflects the degrees of dose conformity and the concentric lamellarity of dose gradients just several millimeters outside and inside the GTV boundary [6,8,18]. The higher the coverage values, the superior the dose conformity and the concentric lamellarity of dose gradients [6,8].

Recording the *D*_eIIV_s and the coverage values of the GTV - 2 mm and GTV - 4 mm for the GTV of <0.72 cc (≤0.33 cc) and <2.20 cc (≤1.26 cc), respectively, were excluded due to the small volumes that were deemed inappropriate for evaluation. Therefore, the dosimetric evaluations for the GTV - 2 mm and GTV - 4 mm were performed on 28 and 26 cases, respectively.

The GTV dose conformity was compared using the smallness of the PIV spillage (cc) outside the GTV and the high GTV coverage value (%) by the *D*_eIIV_ [18]. The steepness of dose gradients outside the GTV and GTV + 2 mm was compared using the smallness of the IIVs of 75 and 50% of the GTV *D*_V-0.01 cc_, excluding the GTV [18,24]. The appropriateness of the dose attenuation margin outside the GTV was compared using the low *D*_eIIV_ of the GTV + 2 mm and the high coverage value of GTV + 2 mm by the *D*_eIIV_ [6]. The steepness of dose increase inside the GTV boundary was compared using the *D*_eIIV_s (%) of GTV, GTV - 2 mm, and GTV - 4 mm [8]. In particular, the GTV *D*_eIIV_ reflects the steepness of dose increase just inside the prescribed isodose surface (IDS), while the higher GTV *D*_eIIV_ compared to the *D*_V-0.01 cc_ is also associated with greater GTV over-coverage by the prescribed dose [18]. Therefore, the closer the prescribed dose (GTV *D*_V-0.01 cc_) and the GTV *D*_eIIV_ are, the better the GTV dose conformity is.

For statistical analyses, paired nonparametric tests were used, considering the distributions of the variables. Box-and-whisker plots (BWPs) were used to represent the distributions of variables. In the BWP, the whiskers denote the nearest values ≤1.5 times the IQR. The cross marks beyond the lines indicate the individual outliers >1.5 times the IQR. The Spearman’s rank correlation coefficient (SRCC) was used to evaluate any correlation between two numerical variables. Friedman’s test (FT) and Scheffe’s post hoc test (SPHT) were used to compare three numerical variables. If there was no significant difference between two numerical variables in the SPHT, Wilcoxon signed-rank test (WSRT) was additionally applied to compare them. In this study, the comparison of different CF selections and settings included the optimizations with or without the Quadratic Overdose CF and those between the 4 cm vs. 8 cm MAT in the Conformality CF, although this study was based on the comparison of three groups. Therefore, any significant differences between the two groups in the WSRT were considered meaningful. Statistical significance was considered at P < 0.05 (*), P < 0.01 (**) and P < 0.001 (***). Statistical analyses were performed using BellCurve for Excel (version 4.05; Social Survey Research Information Co., Ltd., Tokyo, Japan).

## Results

There was a tendency that the larger the GTV, the smaller the depth, although statistically insignificant (Figure 1C). The results of the dosimetric comparison between the three CF selections and settings are shown in Table 2.

**Table 2:** Dosimetric comparison between the three different cost function selections and settings. When comparing two groups, if there was no significant difference in Scheffe’s post hoc test (SPHT), Wilcoxon signed-rank test (WSRT) was added. If there is a significant difference in the comparison between two groups, the respective median values are added. _wo_QO: without Quadratic Overdose; _w_QO_4 cm; with Quadratic Overdose with 4 cm margin around target (MAT); _w_QO_8 cm; with Quadratic Overdose with 8 cm MAT; NS: not significant; PIV: prescribed isodose volume; GTV: gross tumor volume; *D*_eIIV_: the minimum dose to cover the irradiated isodose volume equivalent to a target volume on the dose-volume histogram; GTV + 2 mm: GTV evenly expanded by 2 mm; X% IIV: the volume irradiated with ≥X% of the prescribed dose, including a target volume; *D*_V-0.01 cc_: a minimum dose to cover a target volume minus 0.01 cc; IDS: isodose surface; GTV – X mm: GTV evenly reduced by X mm.

Dosimetric parameters	Friedman’s test (P value)	Scheffe’s post hoc test (Wilcoxon signed-rank test): *P* value [Median values]		
		_wo_QO vs _w_QO_4 cm	_wo_QO vs _w_QO_8 cm	_w_QO_4 cm vs _w_QO_8 cm
Rescaling ratio	<0.001 ***	<0.001 *** [0.976 vs 1.016]	<0.001 *** [0.976 vs 1.016]	0.874 NS (0.626 NS)
PIV spillage (cc)	0.014 *	0.133 NS (0.002 **) [2.41 vs 2.20]	0.017 * [2.41 vs 2.26]	0.701 NS (0.178 NS)
GTV *D*_eIIV_ coverage (%)	< 0.001 ***	<0.001 *** [96.2 vs 96.4]	<0.001 *** [96.2 vs 96.4]	0.806 NS (0.632 NS)
GTV + 2 mm *D*_eIIV_ (%)	< 0.001 ***	<0.001 *** [129.7 vs 134.6]	<0.001 *** [129.7 vs 135.8]	0.812 NS (0.558 NS)
GTV + 2 mm *D*_eIIV_ coverage (%)	< 0.001 ***	<0.001 *** [96.5 vs 96.9]	<0.001 *** [96.5 vs 96.8]	0.618 NS (0.338 NS)
75% IIV spillage (cc)	<0.001 ***	<0.001 *** [10.06 vs 8.52]	<0.001 *** [10.06 vs 8.53]	0.949 NS (0.221 NS)
50% IIV spillage (cc)	<0.001 ***	<0.001 *** [25.01 vs 20.12]	<0.001 *** [25.01 vs 20.42]	1.000 NS (0.241 NS)
*D*_V-0.01 cc_ %IDS (%)	0.048 *	0.049 * [61.4 vs 58.6]	0.365 NS (0.002 **) [61.4 vs 58.0]	0.587 NS (0.600 NS)
GTV *D*_eIIV_ (%)	0.082 NS	0.153 NS (0.013 *) [108.9 vs 109.6]	0.153 NS (0.0495 *) [108.9 vs 109.8]	1.000 NS (0.658 NS)
GTV – 2 mm *D*_eIIV_ (%)	<0.001 ***	<0.001 *** [129.7 vs 134.6]	0.002 ** [129.7 vs 135.8]	0.725 NS (0.699 NS)
GTV – 2 mm *D*_eIIV_ coverage (%)	0.020 *	0.040 * [93.6 vs 94.7]	0.076 NS (0.003 **) [93.6 vs 95.9]	0.965 NS (0.918 NS)
GTV – 4 mm *D*_eIIV_ (%)	<0.001 ***	<0.001 *** [141.9 vs 150.0]	0.002 ** [141.9 vs 152.0]	0.786 NS (0.970 NS)
GTV – 4 mm *D*_eIIV_ coverage (%)	0.064 NS	0.093 NS (0.046 *) [88.0 vs 89.7]	0.940 NS (0.110 NS)	0.188 NS (0.065 NS)

The results of the dosimetric comparison between the _w_QO_4 cm and the _w_QO_8 cm, based on the GTV and its depth, are shown in Table 3.

**Table 3:** Dosimetric comparison between the two groups, the wQO_4 cm vs. wQO_8 cm, based on GTV and its depth. All comparisons were conducted with the WSRT. If there is a significant difference in the comparison between the two groups. _w_QO_4 cm; with Quadratic Overdose with 4 cm margin around target (MAT); _w_QO_8 cm; with Quadratic Overdose with 8 cm MAT; GTV: gross tumor volume; WSRT: Wilcoxon signed-rank test; NS: not significant; PIV: prescribed isodose volume; *D*_eIIV_: the minimum dose to cover the irradiated isodose volume equivalent to a target volume on the dose-volume histogram; GTV + 2 mm: GTV evenly expanded by 2 mm; X% IIV: the volume irradiated with ≥X% of the prescribed dose, including a target volume; *D*_V-0.01 cc_: a minimum dose to cover a target volume minus 0.01 cc; IDS: isodose surface; GTV – X mm: GTV evenly reduced by X mm.

Parameters	GTV: *P* value [Median values]			Depth: *P* value [Median values]	
	<10.00 cc	≥10.00 cc	≥14.00 cc	≤40 mm	≥41 mm
Rescaling ratio	0.900 NS	0.607 NS	0.113 NS	0.975 NS	0.255 NS
PIV spillage (cc)	0.865 NS	0.112 NS	0.033 * [4.09 vs 3.89]	1.000 NS	0.036 * [1.97 vs 1.92]
GTV *D*_eIIV_ coverage (%)	0.593 NS	1.000 NS	0.753 NS	0.008 ** [96.44 vs 96.37]	0.084 NS
GTV + 2 mm *D*_eIIV_ (%)	0.865 NS	0.233 NS	0.033 * [87.5 vs 87.1]	0.776 NS	0.532 NS
GTV + 2 mm *D*_eIIV_ coverage (%)	0.208 NS	0.977 NS	0.326 NS	0.649 NS	0.025 * [96.88 vs 96.94]
75% IIV spillage (cc)	0.910 NS	0.191 NS	0.046 * [18.56 vs 18.46]	0.496 NS	0.334 NS
50% IIV spillage (cc)	0.496 NS	0.100 NS	0.019 * [47.16 vs 46.67]	0.394 NS	0.427 NS
*D*_V-0.01 cc_ %IDS (%)	0.281 NS	0.015 * [59.2 vs 59.6]	0.013 * [59.3 vs 59.8]	0.776 NS	0.307 NS
GTV *D*_eIIV_ (%)	0.427 NS	0.212 NS	0.039 * [110.1 vs 110.0]	0.865 NS	0.307 NS
GTV – 2 mm *D*_eIIV_ (%)	0.553 NS	0.173 NS	0.075 NS	0.594 NS	0.246 NS
GTV – 2 mm *D*_eIIV_ coverage (%)	0.600 NS	0.629 NS	0.753 NS	0.315 NS	0.433 NS
GTV – 4 mm *D*_eIIV_ (%)	0.155 NS	0.125 NS	0.064 NS	0.600 NS	0.507 NS
GTV – 4 mm *D*_eIIV_ coverage (%)	0.0099 ** [86.2 vs 85.6]	0.925 NS	0.917 NS	0.034 * [89.1 vs 90.2]	0.753 NS

Before rescaling the GTV coverage after initial optimization, there was a tendency for the GTV over-coverage in the _wo_QO, while the under-coverages in the _w_QO_4 cm and _w_QO_8 cm (Figure 1D, Table 2). In addition, there was a large variation in the rescaling ratios in the _w_QO_4 cm (Figure 1D).

**Figure 3:**
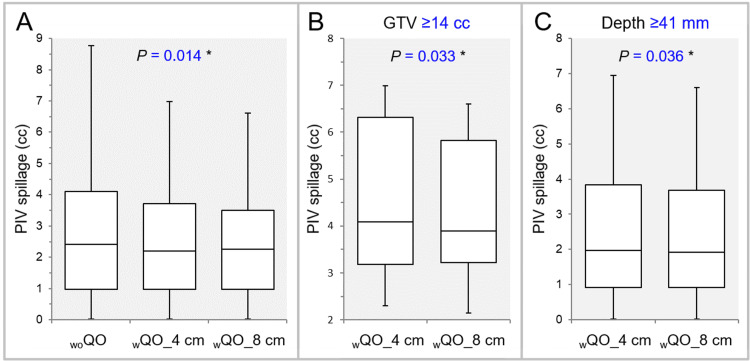
Comparison of GTV dose conformity with prescribed isodose volume spillage outside the GTV. The images show BWPs (A-C), along with the results of FT (A) and Wilcoxon signed-rank test (WSRT) (B,C), for comparisons between the three groups (A), between the _w_QO_4 cm and _w_QO_8 cm limited to GTVs of ≥14 cc (B), and between the _w_QO_4 cm and _w_QO_8 cm limited to the GTVs of ≥41 mm (C). GTV: gross tumor volume; PIV: prescribed isodose volume; _wo_QO: without Quadratic Overdose; _w_QO_4 cm; with Quadratic Overdose with 4 cm margin around target (MAT); _w_QO_8 cm; with Quadratic Overdose with 8 cm MAT; BWPs: box-and-whisker plots; FT: Friedman’s test.

Overall, the GTV dose conformity was significantly superior in the _w_QO_4 cm and _w_QO_8 cm than in the _wo_QO in terms of the small PIV spillage and the high GTV coverage with the *D*_eIIV_, while there was no significant difference between the _w_QO_4 cm and _w_QO_8 cm (Table 2, Figure 3A).

For the GTV of ≥14 cc and the depth of ≥41 mm, the PIV spillage was significantly smaller in the _w_QO_8 cm than in the _w_QO_4 cm (Table 3, Figure 3B,3C). Meanwhile, for the GTV depth of ≤40 mm, the GTV coverage with the *D*_eIIV_ was significantly higher in the _w_QO_4 cm than in the _w_QO_8 cm (Table 3).

**Figure 4:**
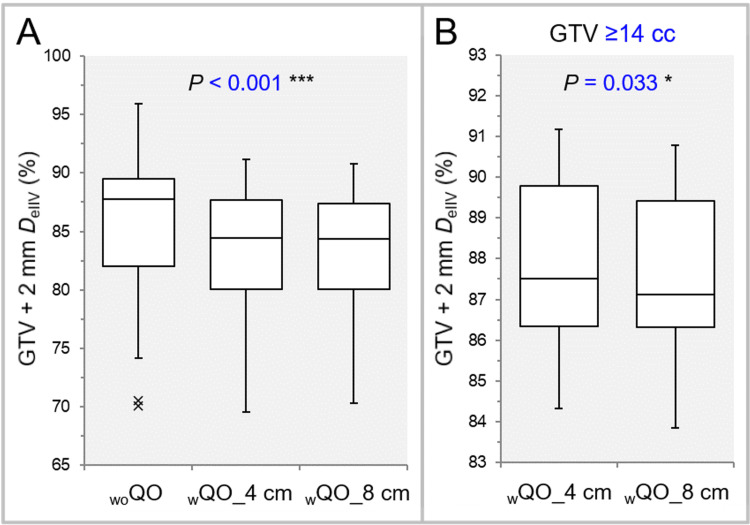
Comparison of the appropriateness of dose attenuation margin outside the GTV using the GTV + 2 mm DeIIV. The images show BWPs (A,B), along with the results of FT (A) and WSRT (B), for comparisons between the three groups (A) and between the _w_QO_4 cm and _w_QO_8 cm limited to GTVs of ≥14 cc (B). The GTV + 2 mm *D*_eIIV_ is shown as a relative value to the GTV *D*_V-0.01 cc_ (100%). GTV: gross tumor volume; GTV + 2 mm: GTV evenly expanded by 2 mm; *D*_eIIV_: the minimum dose to cover the irradiated isodose volume equivalent to a target volume on the dose-volume histogram; _wo_QO: without Quadratic Overdose; _w_QO_4 cm; with Quadratic Overdose with 4 cm margin around target (MAT); _w_QO_8 cm; with Quadratic Overdose with 8 cm MAT; BWPs: box-and-whisker plots; FT: Friedman’s test; WSRT: Wilcoxon signed-rank test; *D*_V-0.01 cc_: a minimum dose to cover a target volume minus 0.01 cc.

The dose attenuation margin outside the GTV was significantly lower and more appropriate in the _w_QO_4 cm and _w_QO_8 cm than in the _wo_QO, while there was no significant difference between the _w_QO_4 cm and _w_QO_8 cm (Table 2, Figure 4A).

For the GTV of ≥14 cc, the dose attenuation margin outside the GTV was significantly lower in the _w_QO_8 cm than in the _w_QO_4 cm (Table 3, Figure 4B).

**Figure 5:**
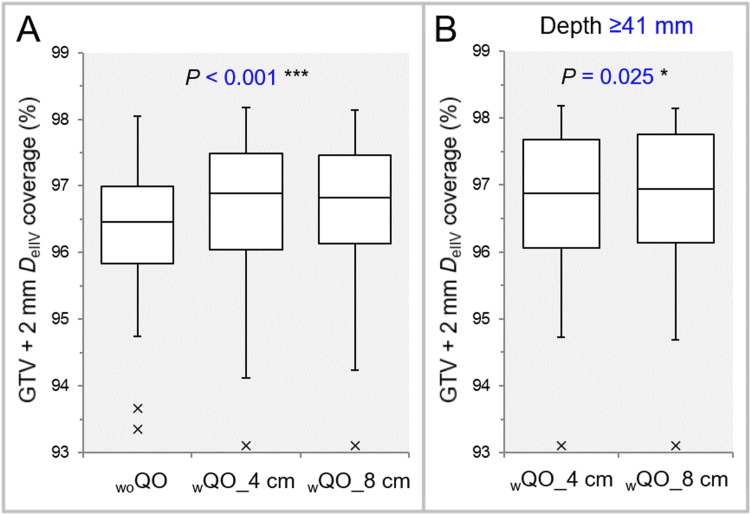
Comparison of the degree of concentric lamellarity of dose gradient outside the GTV using the coverage value of GTV + 2 mm DeIIV. The images show BWPs (A,B), along with the results of FT (A) and WSRT (B), for comparisons between the three groups (A) and between the _w_QO_4 cm and _w_QO_8 cm limited to the GTV depths of ≥41 mm (B). GTV: gross tumor volume; GTV + 2 mm: GTV evenly expanded by 2 mm; *D*_eIIV_: the minimum dose to cover the irradiated isodose volume equivalent to a target volume on the dose-volume histogram; _wo_QO: without Quadratic Overdose; _w_QO_4 cm; with Quadratic Overdose with 4 cm margin around target (MAT); _w_QO_8 cm; with Quadratic Overdose with 8 cm MAT; BWPs: box-and-whisker plots; FT: Friedman’s test; WSRT: Wilcoxon signed-rank test.

The dose conformity to the GTV + 2 mm and the degree of concentric lamellarity of the dose gradient just outside the GTV were superior in the _w_QO_4 cm and _w_QO_8 cm than in the _wo_QO in terms of the GTV + 2 mm coverage with the *D*_eIIV_, while there was no significant difference between the _w_QO_4 cm and _w_QO_8 cm (Table 2, Figure 5A).

For the GTV depth of ≥41 mm, the coverage value of the GTV + 2 mm with the *D*_eIIV_ was significantly higher in the _w_QO_8 cm than in the _w_QO_4 cm (Table 3, Figure 5B).

**Figure 6:**
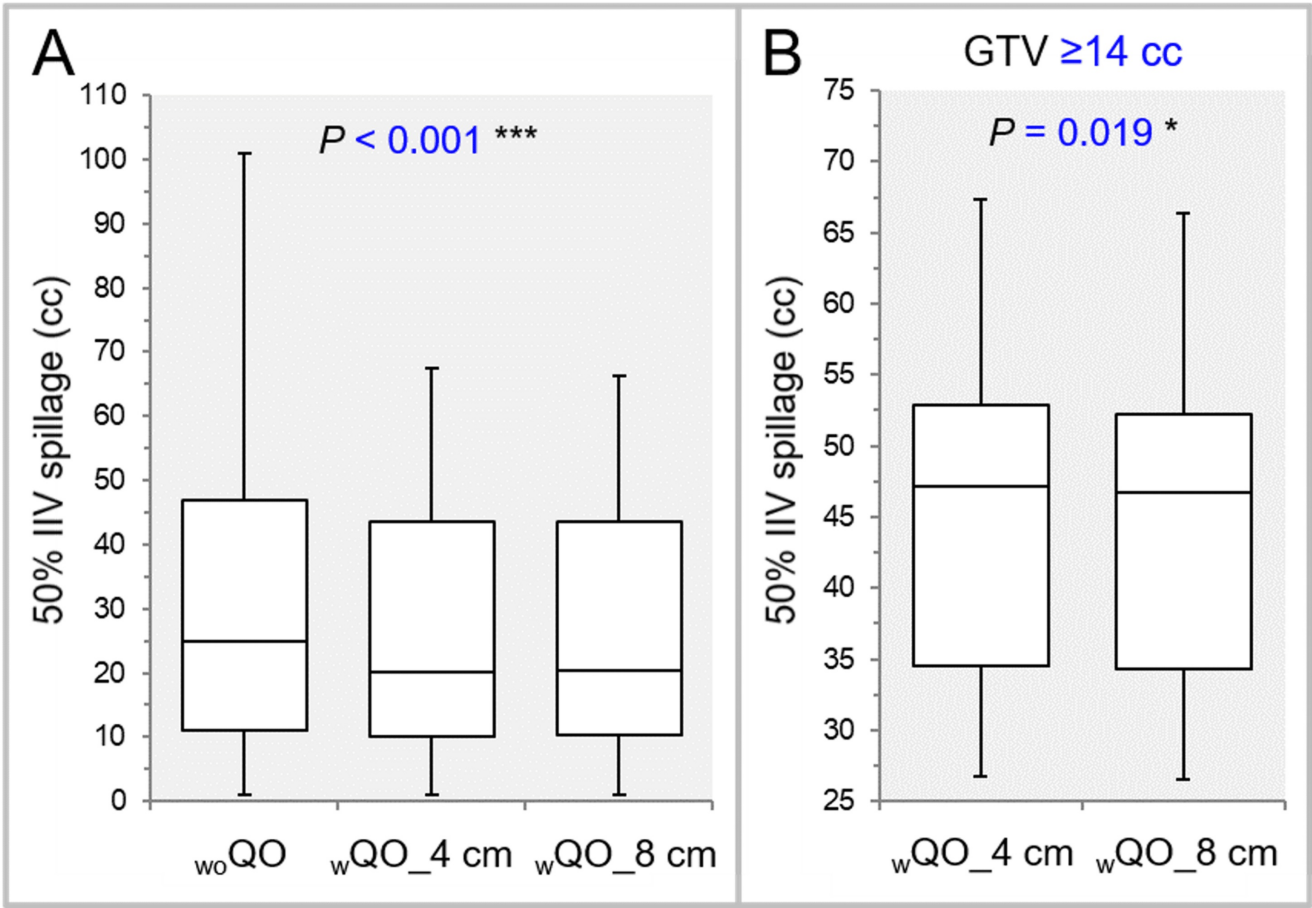
Comparison of the steepness of dose gradient outside the GTV using the volume irradiated with ≥50% of the prescribed dose. The images show BWPs (A,B), along with the results of FT (A) and WSRT (B), for comparisons between the three groups (A) and between the _w_QO_4 cm and _w_QO_8 cm limited to GTVs of ≥14 cc (B). GTV: gross tumor volume; 50% IIV: the volume irradiated with ≥50% of the prescribed dose, including a target volume; _wo_QO: without Quadratic Overdose; _w_QO_4 cm; with Quadratic Overdose with 4 cm margin around target (MAT); _w_QO_8 cm; with Quadratic Overdose with 8 cm MAT; BWPs: box-and-whisker plots; FT: Friedman’s test; WSRT: Wilcoxon signed-rank test.

The dose gradient outside the GTV was significantly steeper in the _w_QO_4 cm and _w_QO_8 cm than in the _wo_QO in terms of the 75% and 50% IIV spillages, while there was no significant difference between the _w_QO_4 cm and _w_QO_8 cm (Table 2, Figure 6A).

For the GTV of ≥14 cc, the dose gradient outside the GTV was significantly steeper in the _w_QO_8 cm than in the _w_QO_4 cm in terms of the 75% and 50% IIV spillages (Table 3, Figure 6B).

**Figure 7:**
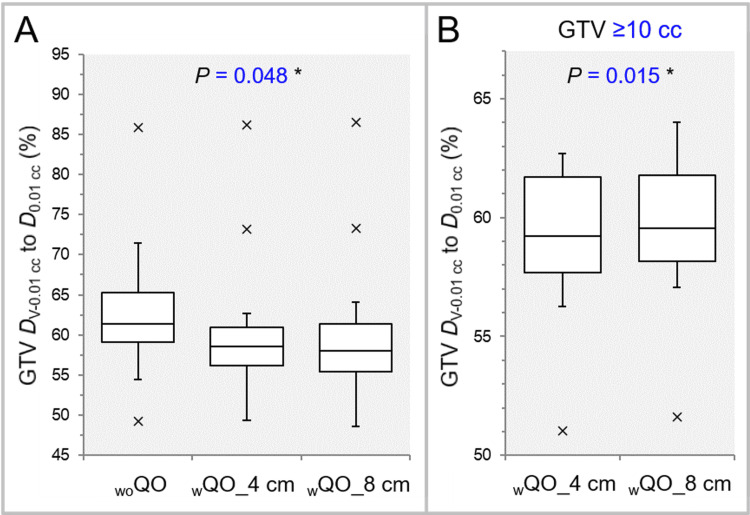
Comparison of the GTV dose inhomogeneity using the GTV DV-0.01 cc relative to the near-maximum dose. The images show BWPs (A,B), along with the results of FT (A) and WSRT (B), for comparisons between the three groups (A) and between the _w_QO_4 cm and _w_QO_8 cm limited to GTVs of ≥10 cc (B). GTV: gross tumor volume; *D*_V-0.01 cc_: a minimum dose to cover a target volume minus 0.01 cc; *D*_0.01 cc_: a minimum dose covering 0.01 cc of a target volume; _wo_QO: without Quadratic Overdose; _w_QO_4 cm; with Quadratic Overdose with 4 cm margin around target (MAT); _w_QO_8 cm; with Quadratic Overdose with 8 cm MAT; BWPs: box-and-whisker plots; FT: Friedman’s test; WSRT: Wilcoxon signed-rank test.

The GTV dose was significantly more inhomogeneous in the _w_QO_4 cm and _w_QO_8 cm than in the _wo_QO, while there was no significant difference between the _w_QO_4 cm and _w_QO_8 cm (Table 2, Figure 7A).

For the GTV of ≥10 cc, the GTV dose was significantly more inhomogeneous in the _w_QO_4 cm than in the _w_QO_8 cm (Table 3, Figure 7B).

**Figure 8:**
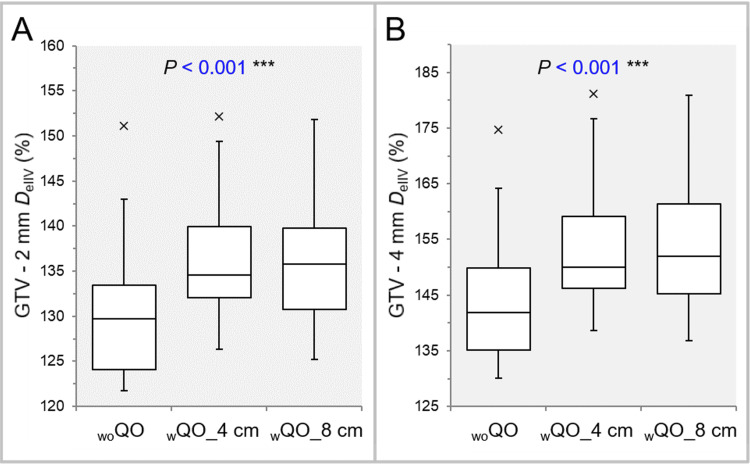
Comparison of the steepness of dose increase inside the GTV using the GTV - 2 mm and GTV – 4 mm DeIIV. The images show BWPs, along with the results of FT, for comparisons between the three groups (A,B). GTV: gross tumor volume; GTV – X mm: GTV evenly reduced by X mm; *D*_eIIV_: the minimum dose to cover the irradiated isodose volume equivalent to a target volume on the dose-volume histogram; _wo_QO: without Quadratic Overdose; _w_QO_4 cm; with Quadratic Overdose with 4 cm margin around target (MAT); _w_QO_8 cm; with Quadratic Overdose with 8 cm MAT; BWPs: box-and-whisker plots; FT: Friedman’s test.

The dose gradient 2-4 mm inside the GTV boundary was significantly steeper in the _w_QO_4 cm and _w_QO_8 cm than in the _wo_QO, while there was no significant difference between the _w_QO_4 cm and _w_QO_8 cm (Tables 2, 3 and Figure 8A, 8B).

**Figure 9:**
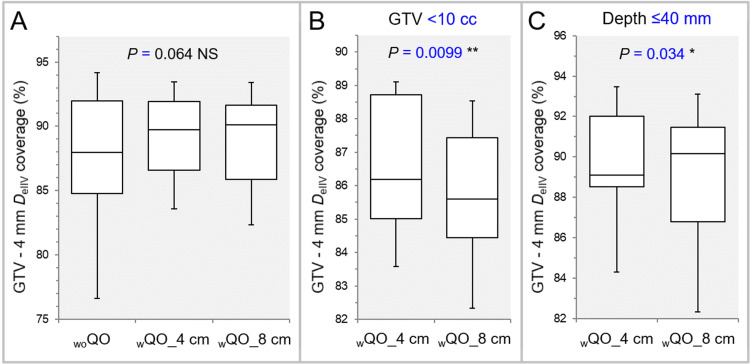
Comparison of the degree of concentric lamellarity of dose gradient inside the GTV using the coverage value of GTV -4 mm DeIIV. The images show BWPs (A,B), along with the results of FT (A) and WSRT (B), for comparisons between the three groups (A), between the _w_QO_4 cm and _w_QO_8 cm limited to GTVs of <10 cc (B), and between the _w_QO_4 cm and _w_QO_8 cm limited to the GTV depths of ≤40 mm (C). GTV: gross tumor volume; GTV - 4 mm: GTV evenly reduced by 4 mm; *D*_eIIV_: the minimum dose to cover the irradiated isodose volume equivalent to a target volume on the dose-volume histogram; _wo_QO: without Quadratic Overdose; _w_QO_4 cm; with Quadratic Overdose with 4 cm margin around target (MAT); _w_QO_8 cm; with Quadratic Overdose with 8 cm MAT; BWPs: box-and-whisker plots; FT: Friedman’s test; WSRT: Wilcoxon signed-rank test.

The degrees of concentric lamellarity at the doses 2 and 4 mm inside the GTV boundary was significantly superior in the _w_QO_4 cm and _w_QO_8 cm than in the _wo_QO, while there was no significant difference between the _w_QO_4 cm and _w_QO_8 cm (Tables 2, 3 and Figure 9A).

For the GTV of <10 cc and the depth of ≤40 mm, the degree of concentric lamellarity at the dose 4 mm inside the GTV boundary was significantly higher in the _w_QO_4 cm than in the _w_QO_8 cm (Table 3, Figure 9B, 9C).

**Figure 10:**
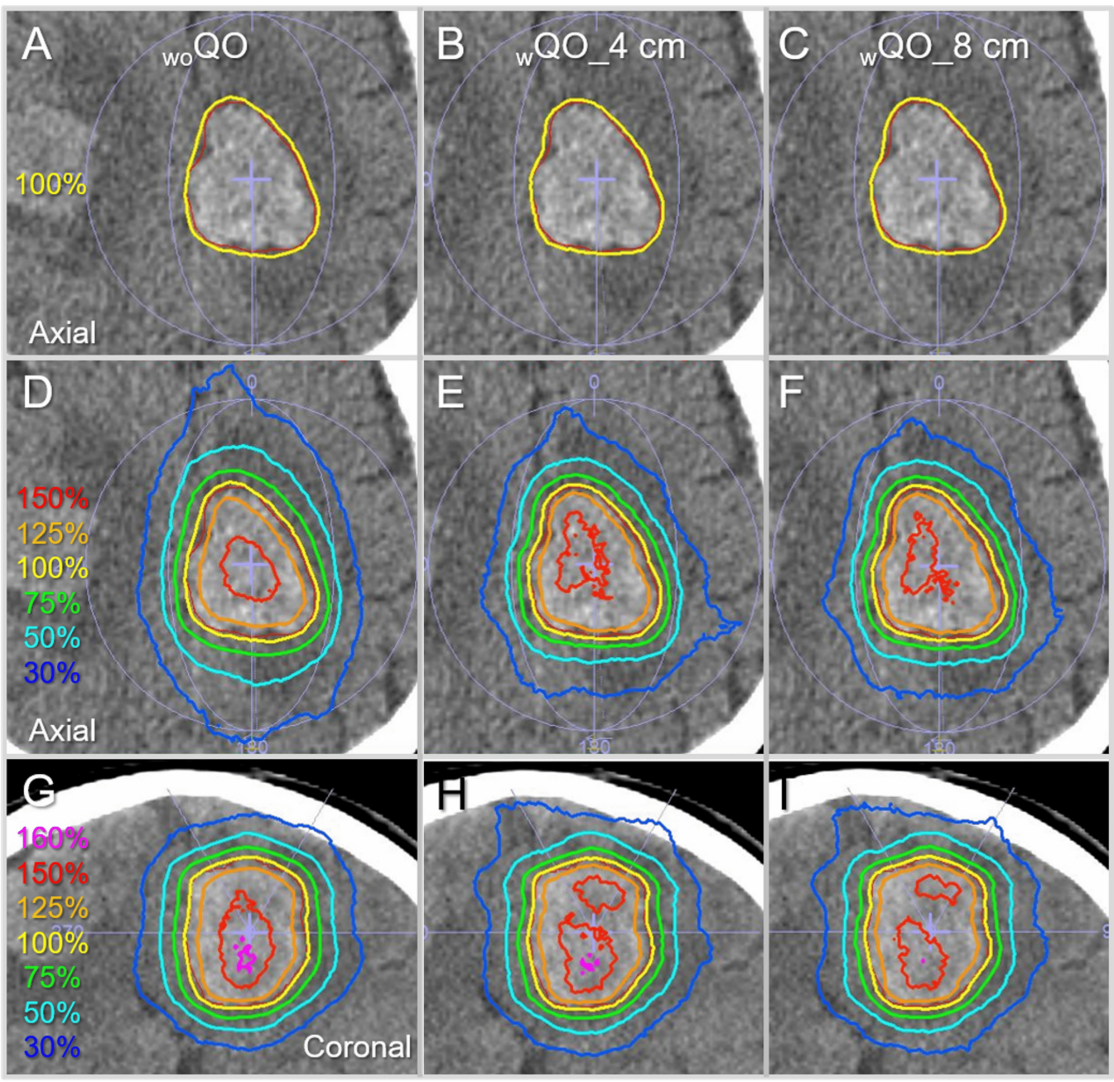
Comparison of dose distributions for single brain metastasis with a GTV of 28.88 cc. The images show head computed tomography images of a patient harboring multiple brain metastases including the lesion in the left fronto-parietal lobe with the GTV of 28.88 cc (A-I), onto which the GTV contoured in red, arc arrangements, and representative isodoses are superimposed; axial views  (A-F); and coronal views (G-I). The isodose lines are shown as relative values with the GTV *D*_V-0.01 cc_ (*D*_99.97%_) as 100% (yellow). _wo_QO: without Quadratic Overdose; _w_QO_4 cm; with Quadratic Overdose with 4 cm margin around target (MAT); _w_QO_8 cm; with Quadratic Overdose with 8 cm MAT; GTV: gross tumor volume; *D*_V-0.01 cc_: a minimum dose covering a target volume minus 0.01 cc.

Figure 10 shows the representative differences in the isodose distributions based on the three different optimizations for the GTV of 28.88 cc.

## Discussion

In the Monaco system, even just applying the Conformality CF enables an efficient dose reduction to normal tissues surrounding a GTV by steepening the dose fall-off outside the GTV boundary from high to low doses [7]. This study revealed that further adding the Quadratic Overdose CF significantly improves the overall dose distributions in comparison to optimization with the Target Penalty and Conformality alone. Specifically, the addition of the Quadratic Overdose significantly enhanced the GTV dose conformity and the steepness of dose gradients both outside and inside the GTV boundary with the degrees of concentric lamellarity [6,8,24]. In this study, the normal tissues to which the Quadratic Overdose is applied was limited and fixed to ≥2 mm outside the GTV boundary with the Shrink Margin option to avoid penalizing voxels within a specified distance from a GTV boundary. However, even with a sufficient margin of 2 mm, the GTV coverage with the assigned dose after initial optimization was generally insufficient, especially in the large lesions. Therefore, the applicable range of the Quadratic Overdose may need to be individually adjusted depending on the GTV. Furthermore, applying the additional Quadratic Overdose to the region ≥5 mm outside the GTV boundary may render the dose gradient even steeper. However, it is difficult to determine the physically reasonable specific dose that should be assigned to as a dose constraint depending on the tumor volume and shape.

Regarding the range of voxels for optimization with the Conformality CF, selecting the MAT option of 8 cm that considers the overall voxels involved in the beam paths significantly improved the dose conformity and gradient outside the GTV for large and deeply located lesions, although the MAT of 8 cm was not always the appropriate choice. Since we adopted the arc arrangement with 180º-360º rotations, optimization considering the overall voxels through which the beams pass may lead to the appropriate optimization. Meanwhile, limiting the voxels to the peri-lesional area with the 4-cm MAT can be beneficial in terms of the steepness of dose increase inside the GTV for some lesions [8,9]. However, from the standpoint of maintaining a GTV marginal dose, the normal tissue dose reduction is prioritized over the GTV dose inhomogeneity. Therefore, optimization of the overall voxels with the MAT of 8 cm is recommended for the underlying CF selection and settings. Taken together, the combination of at least the three physical CF selections and settings described in this study can be a promising candidate for a template for expeditious planning after structure delineation. 

In this study, the grid spacing of 2 mm and the statistical uncertainty of 3% per calculation were the initial settings for XVMC-based dose calculation. The actual time required to complete a single optimization was approximately within 5-10 minutes, allowing prompt planning. Reducing the statistical uncertainty from 3% to ≤1% may further improve dose distribution, while definitely increasing the optimization time [20].

In DCA with either forward or inverse planning, a GTV or PTV dose inhomogeneity usually needs to be predetermined by assigning a specific % IDS (e.g. 70%) for the TV covering relative to the isocenter or maximum dose before planning [5,10,17]. In contrast, in VMA optimization using Monaco, the GTV dose inhomogeneity substantially differs among individual lesions as a result of optimization that prioritizes the steepness of dose gradient outside the GTV. The GTV dose heterogeneity, which is physically advantageous for steepening the dose gradient outside the GTV boundary, varies depending on tumor volume, shape, depth, and/or presence of other nearby lesions and is never constant. Thus, for producing the dose distribution suitable for each lesion, VMA optimization with CFs like the Conformality seems to be more rational than DCA that needs to predefine the target dose heterogeneity to a certain constant value.

This planning study inevitably has several limitations inherent to the study design limited only to the process of SRS planning using a certain system. The rigorous comparisons of the times required for VMA optimization and actual irradiation were not included. The consistency and robustness between the planned dose distribution and actual dose verification were also not confirmed. The most important issue is whether the CF combination based on the results of this study actually contributes to improving treatment outcomes. This study was also limited to irradiation of a single lesion. When irradiating multiple lesions with a single isocenter, even if the same optimization method is applied, the resulting dose distributions may vary considerably due to the dose interference. Furthermore, even with the same CF selection and settings, the dose distribution after optimization can differ if the leaf width or configuration of MLC differ. For example, the dose attenuation outside a GTV can be steeper in a high-definition MLC with the leaf width of 2.5 mm than the Agility MLC [5]. In this study, we focused on the three physical CFs among three biological and five physical CFs available in Monaco. It remains unclear whether better dose distribution can be achieved by leveraging biological CFs [19]. It is hoped that other facilities using Monaco will propose more useful CF selection and settings based on different perspectives.

## Conclusions

With VMA optimization by applying just three physical CFs - Target Penalty, Conformality, and Quadratic Overdose - to a GTV and the head surface contour, Monaco can efficiently produce dose distributions suitable for SRS of single BMs of various sizes and shapes, even for a 5-mm leaf-width MLC mounted in a standard linac. For efficiently minimizing the dose outside the GTV boundary from high to low doses, optimizing the overall voxels around the GTV is suitable, especially for large and deeply located lesions. Templating the combination of the three CFs with the detailed settings allows for semi-automated and rapid treatment planning, leading to the prompt start of irradiation after image acquisition, e.g. within 24 hours.
